# The Impact of COVID-19 Quarantine on Tuberculosis and Diabetes Mellitus Cases: A Modelling Study

**DOI:** 10.3390/tropicalmed7120407

**Published:** 2022-11-29

**Authors:** Nuning Nuraini, Ilham Saiful Fauzi, Bony Wiem Lestari, Sila Rizqina

**Affiliations:** 1 Department of Mathematics, Faculty of Mathematics and Natural Sciences, Institut Teknologi Bandung, Bandung 40132, Indonesia; 2 Center for Mathematical Modeling and Simulation, Institut Teknologi Bandung, Bandung 40132, Indonesia; 3 Department of Accounting, Politeknik Negeri Malang, Malang 65141, Indonesia; 4 Department of Public Health, Faculty of Medicine, Universitas Padjadjaran, Bandung 40161, Indonesia; 5 Department of Internal Medicine, Radboud Institute for Health Sciences, Radboud University Medical Centre, 6525 GA Nijmegen, The Netherlands

**Keywords:** COVID-19, tuberculosis, diabetes, mathematical model, quarantine, control strategy

## Abstract

COVID-19 has currently become a global pandemic and caused a high number of infected people and deaths. To restrain the coronavirus spread, many countries have implemented restrictions on people’s movement and outdoor activities. The enforcement of health emergencies such as quarantine has a positive impact on reducing the COVID-19 infection risk, but it also has unwanted influences on health, social, and economic sectors. Here, we developed a compartmental mathematical model for COVID-19 transmission dynamic accommodating quarantine process and including tuberculosis and diabetic people compartments. We highlighted the potential negative impact induced by quarantine implementation on the increasing number of people with tuberculosis and diabetes. The actual COVID-19 data recorded in Indonesia during the Delta and Omicron variant attacks were well-approximated by the model’s output. A positive relationship was indicated by a high value of Pearson correlation coefficient, r=0.9344 for Delta and r=0.8961 for Omicron with a significance level of p<0.05. By varying the value of the quarantine parameter, this study obtained that quarantine effectively reduces the number of COVID-19 but induces an increasing number of tuberculosis and diabetic people. In order to minimize these negative impacts, increasing public awareness about the dangers of TB transmission and implementing a healthy lifestyle were considered the most effective strategies based on the simulation. The insights and results presented in this study are potentially useful for relevant authorities to increase public awareness of the potential risk of TB transmission and to promote a healthy lifestyle during the implementation of quarantine.

## 1. Introduction

Since 2020, the coronavirus disease 2019 (COVID-19) pandemic has impacted countries globally [1,2,3,4]. As of 11 November 2022, about 630 million cases and 6.5 million deaths have been reported [5]. The unprecedented infection and mortality rates have led to the implementation of various public health measures, i.e., lockdowns and social distancing regulations. While these efforts are well-intended to break the chain of transmission, several deleterious health and socioeconomic consequences have been observed [6].

In the field of tuberculosis (TB) management, the COVID-19 pandemic has reversed decades of progress toward TB eradication. Quarantines and stay-at-home measures have increased the risk of TB transmission, particularly among household members [7,8]. This is reflected by the findings from Aznar et al. [9] that observed a significant increase in active TB cases among household contacts in 2020, when compared to 2019. Given that the pandemic also causes a general decrease in healthcare access and increasing poverty rates, both known TB determinants. The number of tuberculosis incidence and mortality are projected to increase by 5–15% within the next five years. This equals hundreds of thousands of additional TB deaths globally [7,8].

The dire situation is potentially exacerbated by comorbidities resulting from quarantine-related lifestyle changes. Several systematic reviews reported that globally, the COVID-19 quarantine measures have led to less physical activity, poorer diet, and a more sedentary lifestyle, all of which are risk factors for many comorbidities, including diabetes [6,10]. In addition, another systematic review reported that lockdowns are associated with deteriorating glycemic control among patients with type-2 diabetes mellitus [11]. Uncontrolled diabetes is a known risk factor for TB infections, poor treatment outcomes, and mortality. In six countries with the highest TB cases, about 10–18% of TB cases can be attributed to diabetes. Overall, diabetes increases the risk of active TB by two- to four-fold [12]. Despite this relationship between TB, diabetes, and COVID-19, however, no study has attempted to model the impact of the pandemic on TB-DM co-occurrence and case management.

This paper aims to investigate the impacts of lockdowns and regional quarantines during the COVID-19 pandemic on TB-DM patients through a modelling study in Indonesia, a country with high burden of TB. Here, we established a mathematical model that divides human population into compartments of three diseases: COVID-19, tuberculosis, and diabetes mellitus. The unobserved parameters were estimated to obtain the best data fitting between the COVID-19 data and the model’s output that accommodates the quarantine process. The actual data used in this study is a weekly report of the COVID-19 cases in Indonesia during June 2021-September 2021 and December 2021-April 2022, when Indonesia experienced the Delta and Omicron variant attacks, respectively. The Indonesian government decided to implement the emergency social activity restriction (PPKM darurat) during these periods. In addition, we proposed some strategies that play essential roles in both limiting COVID-19 infection and reducing the negative impacts on TB-DM patients.

## 2. Material and Methods

### 2.1. Mathematical Model

In our mathematical model, we made some modifications to the standard disease transmission model that describes the dynamic of infection. The model includes compartments of three diseases: COVID-19, tuberculosis, and diabetes. TB and diabetes were selected in this model because of the significant impact of the COVID-19 pandemic on TB medical treatment and diabetes progression, especially during the implementation of quarantine and public activity restriction. TB is an infectious disease whose risk of transmission increases during home quarantine among household members. Meanwhile, diabetes is not a contagious disease but it is a co-morbid disease that increases the mortality rate of COVID-19 patients. Public and social measures that restrict residents’ activities at home possibly change lifestyles that implicate the development of diabetes.

**Figure 1:**
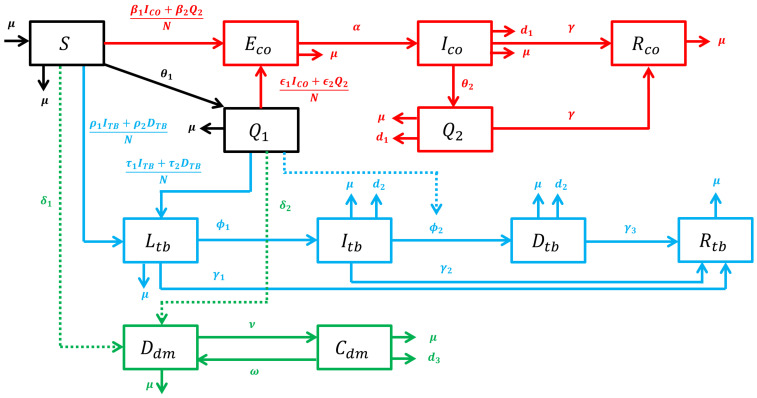
A schematic diagram of the disease transmissions including COVID-19, tuberculosis, and diabetes mellitus. The dashed line indicates the indirect effect of the quarantine implementation.

The actual problem is complex and complicated, so we establish a model that simplifies it by considering only the basic and essential compartments for COVID-19, TB, and diabetes. The human population (N) was divided into twelve compartments: susceptible (S); quarantined susceptible $\left(Q_{1}\right)$; exposed coronavirus $\left(E_{co}\right)$ i.e., individuals were recently exposed by coronavirus and not infectious; infected coronavirus $\left(I_{co}\right)$ i.e., individuals were infected and infectious; quarantined infected coronavirus $\left(Q_{2}\right)$ i.e., individuals were infected, infectious, and treated; recovered coronavirus $\left(R_{co}\right)$ i.e., individuals were recovered from coronavirus infection; latent tuberculosis $\left(L_{tb}\right)$ i.e., individuals were recently exposed tuberculosis and not infectious; infected tuberculosis $\left(I_{tb}\right)$ i.e., individuals were active TB and infectious; diagnosed tuberculosis $\left(D_{tb}\right)$ i.e., individuals were active TB and treated; recovered tuberculosis $\left(R_{tb}\right)$ i.e., individuals were recovered from tuberculosis infection; diabetes without complications $\left(D_{dm}\right)$; and diabetes with complications $\left(C_{dm}\right)$. The complete transmission process is shown as transfer diagram in Figure 1.

The resulting transmission model is given by the following nonlinear ordinary differential equations system:

(1) $$\begin{array}{cc} \; & \frac{dS}{dt}=\mu N-\left(\frac{\beta_{1}I_{co}+\beta_{2}Q_{2}}{N}\right)S-\left(\frac{\rho_{1}I_{tb}+\rho_{2}D_{tb}}{N}\right)S-\left(\delta_{1}+\theta_{1}+\mu \right)S\\ \; & \frac{dQ_{1}}{dt}=\theta_{1}S-\left(\frac{\epsilon_{1}I_{co}+\epsilon_{2}Q_{2}}{N}\right)Q_{1}-\left(\frac{\tau_{1}I_{tb}+\tau_{2}D_{tb}}{N}\right)Q_{1}-\left(\delta_{2}+\mu \right)Q_{1}\\ \; & \frac{dE_{co}}{dt}=\left(\frac{\beta_{1}I_{co}+\beta_{2}Q_{2}}{N}\right)S+\left(\frac{\epsilon_{1}I_{co}+\epsilon_{2}Q_{2}}{N}\right)Q_{1}-\left(\alpha +\mu \right)E_{co}\\ \; & \frac{dI_{co}}{dt}=\alpha E_{co}-\left(\theta_{2}+\gamma +d_{1}+\mu \right)I_{co}\\ \; & \frac{dR_{co}}{dt}=\gamma \left(I_{co}+Q_{2}\right)-\mu R_{co}\\ \; & \frac{dQ_{2}}{dt}=\theta_{2}I_{co}-\left(\gamma +d_{1}+\mu \right)Q_{2}\\ \; & \frac{dL_{tb}}{dt}=\left(\frac{\rho_{1}I_{tb}+\rho_{2}D_{tb}}{N}\right)S+\left(\frac{\tau_{1}I_{tb}+\tau_{2}D_{tb}}{N}\right)Q_{1}-\left(\phi_{1}+\gamma_{1}+\mu \right)L_{tb}\\ \; & \frac{dI_{tb}}{dt}=\phi_{1}L_{tb}-\left(K\phi_{2}+\gamma_{2}+d_{2}+\mu \right)I_{tb}\\ \; & \frac{dD_{tb}}{dt}=K\phi_{2}I_{tb}-\left(\gamma_{3}+d_{2}+\mu \right)D_{tb}\\ \; & \frac{dR_{tb}}{dt}=\gamma_{1}L_{tb}+\gamma_{2}I_{tb}+\gamma_{3}D_{tb}-\mu R_{tb}\\ \; & \frac{dD_{dm}}{dt}=\delta_{1}S+\delta_{2}Q_{1}+\omega C_{dm}-\left(\nu +\mu \right)D_{dm}\\ \; & \frac{dC_{dm}}{dt}=\nu D_{dm}-\left(\omega +d_{3}+\mu \right)C_{dm} \end{array}$$

The recruitment rate of susceptible is equal to human average life expectancy, $\mu_{h}$. Compartments $Q_{1}$ and $Q_{2}$ are the additional compartments to accommodate the implementation of the COVID-19 quarantine. Persons in compartment *S* are transferred into $Q_{1}$ with quarantine rate $\theta_{1}$ and persons in compartment $I_{co}$ are transferred into $Q_{2}$ with quarantine rate $\theta_{2}$. Quarantined susceptibles $Q_{1}$ are still possible to be infected by coronavirus when they have a contact with $I_{co}$ and $Q_{2}$, but at a different infection rate with susceptible *S*. Persons in compartment $Q_{1}$ can be transferred into latent tuberculosis $L_{tb}$ when they are in a close contact with infectious and active TB individuals, $I_{tb}$ or $D_{tb}$, during the quarantine at home. The portion of susceptibles (S) who develop diabetes without complication is $\delta_{1}$ which is shown by green dashed lines in Figure 1 considering that diabetes is not an infectious disease. Persons in compartment $Q_{1}$ also can develop diabetes as an indirect effect of quarantine through lifestyle changes and be transferred into compartment $D_{dm}$ with portion $\delta_{2}$. In addition, people with diabetes who initially do not expose a complication can progress into diabetes with complications $\left(C_{dm}\right)$ at rate ν, and diabetic people who recover from complications are assumed to still suffer from diabetes with rate ω.

There are disease-related deaths caused by the COVID-19 with rate $d_{1}$, tuberculosis with rate $d_{2}$, and diabetes with rate $d_{3}$, then we assumed that the total population *N* is not constant. The dynamic of the total population *N* is given by the following equation:

$$\frac{dN}{dt}=\mu \left(N-X\right)-d_{1}\left(I_{co}+Q_{2}\right)-d_{2}\left(I_{tb}+D_{tb}\right)-d_{3}C_{dm}$$

where $X=S+Q_{1}+E_{co}+I_{co}+R_{co}+Q_{2}+L_{tb}+I_{tb}+D_{tb}+R_{tb}+D_{dm}+C_{dm}$.

Furthermore, to accommodate the inability of health center to optimally diagnose and treat the infected tuberculosis during the implementation of quarantine, we defined a constant *K* influencing the diagnosis rate $\phi_{2}$ as follow:

$$K=\left\{\begin{array}{cc} 1 & \mathrm{if}\;\theta_{1}=0\\ 0.5 & \mathrm{if}\;0<\theta_{1}\leq 1 \end{array}\right.$$

In this model, the population change proportion for each compartment is described by the dynamic of equation system (1). The descriptions and values of each parameter are shown in Table A1 for the fixed parameter values.

### 2.2. Data Fitting

The raw data used in the present study is a weekly recorded COVID-19 cases. The mathematical model will be fitted to a data recorded by Indonesian Health Ministry during the Delta variant and Omicron variant attacks. The Delta variant of the coronavirus has been detected in Indonesia since early June 2021 and the high cases were reported in July 2021 to August 2021, followed by high number of deaths. Indonesia experienced the third wave of the COVID-19 infection with the Omicron variant of the coronavirus in mid-December 2021 to April 2022. The Indonesian government announced a plan to implement the emergency social activity restriction (PPKM darurat) in early July 2021 and mid-January 2022 to anticipate the worst possible consequences caused by the infection of these two variants. Infected corona patients become the priority to get medical treatment during the implementation of quarantine to reduce the number of viral transmission as soon as possible. The inability of health centers to provide optimal services during quarantine induces the other acute health threats to not be handled and treated properly.

The values of unobserved parameter, $\left(\beta_{1},\beta_{2},\epsilon_{1},\epsilon_{2},\rho_{1},\rho_{2},\tau_{1},\tau_{2},\delta_{1},\delta_{2},\phi_{2}\right)$, were estimated by minimizing error between the result of numerical simulation and the actual data. We used Spiral Dynamics Optimization (SDO) method developed by Tamura and Yasuda [13] to minimize root-mean-square error (RMSE) between the data of infected COVID-19 and the model output $\left(I_{co}+Q_{2}\right)$. Further, we implemented 100 bootsrap realizations to obtain the values of parameter with 95% confidence interval. The values of the remaining parameters were obtained from the literature and the references were cited therein (see Table 1 for the value of the fixed parameters).

The initial value of the total population, N(0), approximates the total population in Indonesia at 270 million people. The initial values of COVID-19 compartments were obtained from data retrieved from https://www.covid19.go.id (accessed on 10 July 2022) [14] in the first week of June 2021 for Delta period and third week of December 2021 for Omicron period. In early June 2021, the number of infected was approximately 40 thousand and the number of recovered was 1.7 million, while in mid-December 2021, there were 1287 infected people and around 4.1 milion people recovered from the coronavirus infection. We assumed that only 25% of infected people are quarantined in hospital to receive medical treatment. The number of people exposed by coronavirus but not infectious were considered to be 100 thousand in first week of June 2021 and 50 thousand in third week of December 2021. Using the information obtained from https://www.tbindonesia.or.id (accessed on 10 July 2022) [15], we set the initial value $I_{tb}\left(0\right)=200,000$ and $D_{tb}\left(0\right)=150,000$ at the beginning of Delta period. In the numerical simulation, we also used the initial value $L_{tb}\left(0\right)=500,000$ and $R_{tb}\left(0\right)=2,000,000$. The number of people with diabetes in Indonesia is about 10.8 million, and we assumed that about 25 percent of diabetics have complications. For the Omicron period in mid-December 2021, the initial values were adjusted with some increases from the Delta period.

Furthermore, to accomodate the implementation of quarantine by government, we defined parameter $\theta_{1}$ that denotes the quarantine rate of susceptible who stay at home to restrict social interaction, and parameter $\theta_{2}$ that represents the quarantine rate of coronavirus infected people who get medical treatment from health services during the pandemic. In this research, the quarantine refers to public and social measures that restrict people’s movements and isolate them at home. The implementation of emergency social activity restriction (PPKM darurat) by Indonesian government was considered as the macro quarantine, because it restricted most of non-critical public activities. We assumed that the higher the quarantine level, the lower the ability of health services to accommodate and provide medical treatment for infected people due to the increased number of hospital visits during the COVID-19 pandemic. Further, the value of parameter $\theta_{2}$ decreases when the quarantine was implemented. Table 1 shows the value of quarantine parameters used in numerical simulation for three quarantine scenarios.

### 2.3. Control Strategies

Some continuous controls were modelled by adding a reduction or addition, $u_{i}\left(t\right)$ where *t* represents time in weekly unit, in the differential equation of state related to the controls. The proposed control strategies in this work help to reduce the risk of tuberculosis infection and the risk of diabetes developement during the implementation of quarantine. We added to the mathematical model three control functions $\left(u_{1}\left(t\right),u_{2}\left(t\right),u_{3}\left(t\right)\right)$ associated to tuberculosis interventions and two control functions $\left(u_{4}\left(t\right),u_{5}\left(t\right)\right)$ related to diabetes interventions. The interpretation of each control is given as follow:

1. Control $u_{1}\left(t\right)$: proportion of awareness program for quarantined susceptible to restrict the interaction with tuberculosis suspects in the environment.
2. Control $u_{2}\left(t\right)$: proportion of awareness program for latent tuberculosis by intensifying the latent identification and putting under treatment.
3. Control $u_{3}\left(t\right)$: proportion of diagnosis program for infected tuberculosis by managing a specific team for diagnosis or optimizing the use of telemedicine.
4. Control $u_{4}\left(t\right)$: proportion of awareness program for quarantined susceptible by implementing healthy lifestyle and exercising inside the house.
5. Control $u_{5}\left(t\right)$: proportion of awareness program for diabetic people without complications by applying healthy diet and diet tracking in quarantine period.

The resulting states completed by the controls for each compartment are given by the equations shown in Appendix A.2. In the numerical simulation, we also combined controls $\left(u_{1},u_{2},u_{3}\right)$ to reduce the risk of tuberculosis and controls $\left(u_{4},u_{5}\right)$ to minimize the risk of diabetes. We measured the efficacy of each control strategy by calculating the percentage of reduced cases for some noticed compartments during the time observation.

## 3. Results

In this section, we present the result of numerical simulation using the estimated parameter values that minimize error between the actual COVID-19 data during the Delta and Omicron variant outbreaks with the model’s output. The actual COVID-19 data is data that indicates the weekly number of people infected with COVID-19 recorded by Indonesian government. The output of mathematical model refers to the result of numerical simulation that shows the number of COVID-19 infected people, $I_{co}+Q_{2}$, in a week. We examined the effect of quarantine implementation by comparing the dynamic of TB and diabetes compartments in three scenarios: no quarantine, micro quarantine, and macro quarantine. In addition, we suggested the implementation of some control strategies to reduce the risk of tuberculosis transmission and diabetes development in quarantine period. We considered the variations of control parameter separately, and interpreted the simulation’s result for each proposed control.

### 3.1. Numerical Simulation of Mathematical Model Accomodating Quarantine Process

**Figure 2:**
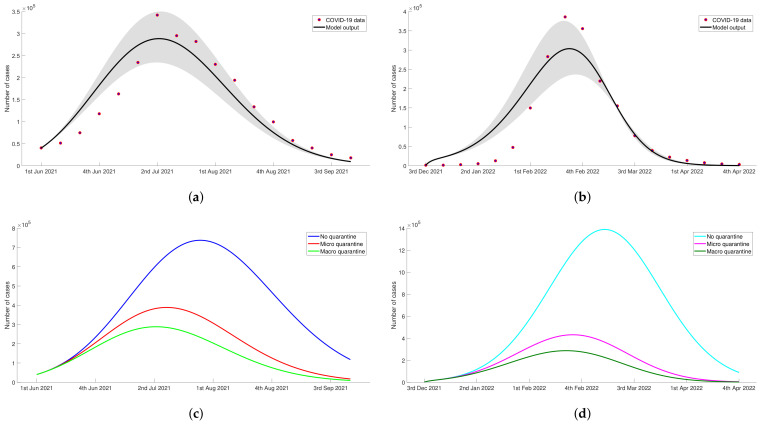
The results of data fitting between the actual COVID-19 data in Indonesia and the output of model during the period of Delta and Omicron variant were shown in (**a**,**b**), respectively. Further, figure (**c**,**d**) show the number of COVID-19 infected people, $I_{co}+Q_{2}$, based on the simulation results of some quarantine scenarios when Indonesia experienced Delta and Omicron variant attacks. (**a**) Data fitting during Delta period. (**b**) Data fitting during Omicron period. (**c**) The number of COVID-19 infected people for each quarantine scenario during Delta period. (**d**) The number of COVID-19 infected people for each quarantine scenario during Omicron period.

The weekly data of COVID-19 cases during the observation time was fitted with the output of mathematical model to obtain the estimated parameters. Table 2 displays the estimated values for each unobserved parameter with 95% confidence interval obtained from 100 bootstrap realizations. As can be seen in Figure 2a,b, the results of simulation produced a good data fitting in both of Delta period and Omicron period. In order to assess the goodness of fit, we calculated the Pearson correlation coefficient between the actual data and the model’s output, denoted by coefficient $r_{d}$ for Delta and $r_{o}$ for Omicron. The calculation yielded $r_{d}=0.9343$ and $r_{o}=0.8961$ with significance level p<0.05, indicating a strong positive relationship between data and simulation result.

For the Delta period, the actual data and model’s output indicate same period of infection peak, the second week of July 2021. The highest number of COVID-19 cases shown in the recorded data was 341,749, while the model resulted 288,169 cases at the peak of infection. The number of COVID-19 cases started to decline significantly in the subsequent weeks. For the Omicron period, the peak of infection shown by data and model’s output is in the third week of February 2022. The number of infected people on this infection peak that recorded in actual data is 385,769 cases, whereas the simulation result indicates that the potential highest case number is only 286,566 cases.

We used the estimated parameter values in Table 2 to simulate the dynamic of TB and diabetes compartments in three scenarios. In Figure 2c,d, we observed that the number of COVID-19 infected people, $\left(I_{co}+Q_{2}\right)$, decreased when the quarantine was implemented during the Delta and Omicron variant outbreaks. The implementation of quarantine not only reduced positive cases but also led to the early occurrence of infection peak; thereby, the emergency period lasted shorter. The summary of simulation results without or with accomodating the implementation of quarantine was given in Table 3. The implementation of micro quarantine reduced 51.48 percent of COVID-19 cases in the Delta period and 69.76 percent in the Omicron period. As expected, a higher decrease in the number of COVID-19 cases was resulted from the implementation of macro quarantine that is 64.17% and 79.60% for the Delta and Omicron period, respectively. When micro and macro quarantine were implemented in Delta period, the number of infected individuals on the peak of infection were 47.40% and 60.76%, respectively, lower than no quarantine scenario. In Omicron period, the percentages of case reduction in the infection peak affected by micro and macro quarantine implementation were 69.19% and 79.27%. The peak of infection shifted two weeks later when the quarantine was not implemented. These implied that quarantine enforced by the government could significantly limiting the spread of COVID-19 infection, particularly during the Delta and Omicron variant outbreaks.

**Figure 3:**
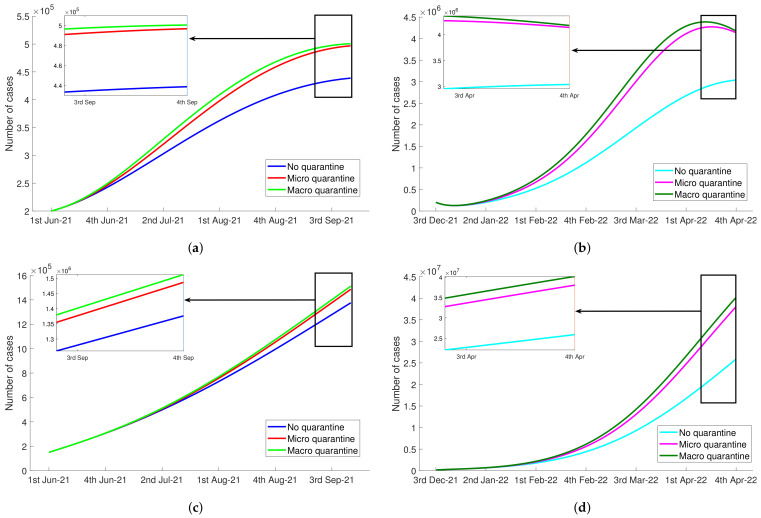
The negative impact of quarantine during Delta variant and Omicron variant of the COVID-19 pandemic to the increasing number of tuberculosis infected people, $I_{tb}$ and $D_{tb}$. (**a**) The number of $I_{tb}$ during Delta period. (**b**) The number of $I_{tb}$ during Omicron period. (**c**) The number of $D_{tb}$ during Delta period. (**d**) The number of $D_{tb}$ during Omicron period.

In Table 2, we observed that infection rate parameters of quarantined susceptible by contact with infected and diagnosed TB, $\tau_{1}$ and $\tau_{2}$, were higher than the infection rates of susceptible, $\rho_{1}$ and $\rho_{2}$, in both the Delta and Omicron periods. This implied a higher number of tuberculosis cases during the quarantine. Figure 3 illustrates how micro and macro quarantine increase the number of TB cases, $I_{tb}$ and $D_{tb}$. We considered three values for $\theta_{1}:0,0.3$, and 0.75. We noticed that as the quarantine rate $\left(\theta_{1}\right)$ increases, the number of $I_{tb}$ and $D_{tb}$ increase. When micro and macro quarantine were implemented during Delta variant outbreak, the number of active infected TB were 13.24% and 14.09%, respectively, higher than the number $I_{tb}$ of no quarantine scenario at the end of observation time. Similar results were also seen in the Omicron period where $I_{tb}$ during micro and macro quarantine scenarios were 36.18% and 37.5% higher than no quarantine. The significant increase in the number of compartment $I_{tb}$ led to the increment in the number of tuberculosis diagnosed individuals despite the diagnosis ability of health services decreased during pandemic. In the last week of September 2021, the number of diagnosed TB of micro and macro quarantine were 8.04% and 9.91%, respectively, higher than the scenario of no implementation of quarantine. In the last week of April 2022, there were 47.10% and 55.21% higher potential diagnosed TB for micro and macro quarantine, respectively. Table 4 displays the summary of quarantine effect on the increasing number of people with tuberculosis and diabetes during the observation time, the Delta and Omicron periods.

**Figure 4:**
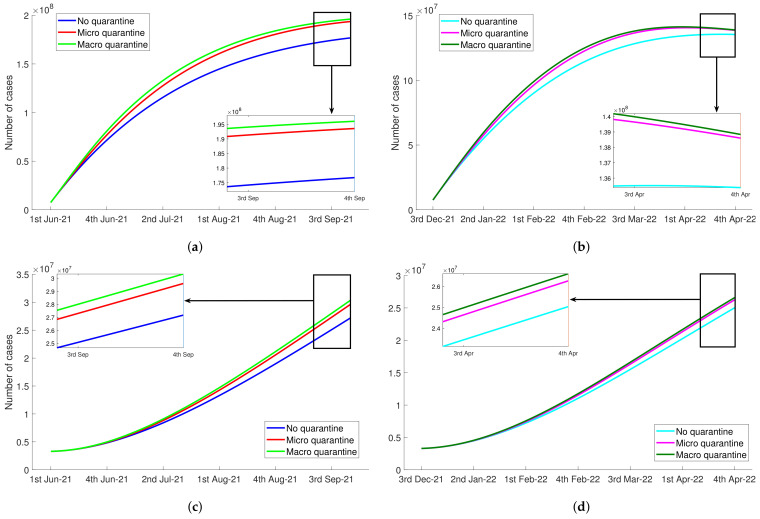
The negative impact of quarantine during Delta variant and Omicron variant of the COVID-19 pandemic to the increasing number of people with diabetes, $D_{dm}$ and $C_{dm}$. (**a**) The number of $D_{dm}$ during Delta period. (**b**) The number of $D_{dm}$ during Omicron period. (**c**) The number of $C_{dm}$ during Delta period. (**d**) The number of $C_{dm}$ during Omicron period.

In Table 2, the probability of quarantined susceptible developing diabetes, $\delta_{2}$, was higher than the probability of susceptible developing diabetes, $\delta_{1}$. This indicated that the implementation of quarantine possibly caused the increase in the number of people with diabetes. We presented the effect of quarantine rate $\theta_{1}$ to the number of diabetic without complication $\left(D_{dm}\right)$ and diabetic with complications $\left(C_{dm}\right)$ in Figure 4. Here, we also considered three values of susceptible quarantine rate: $\theta_{1}=0$ (no quarantine), $\theta_{1}=0.3$ (micro quarantine), and $\theta_{1}=0.75$ (macro quarantine). We observed that the stricter quarantine implementation, that was, greater values for $\theta_{1}$, the higher number of individuals developing diabetes (see Table 4 for the number of people with diabetes without and with complications). More precisely, at the end of Delta variant observation time, micro quarantine increased the number of diabetes without complications 9.53% higher than no quarantine scenario, and macro quarantine led 10.93% increased cases. On the other hand, the number of diabetes with complications in the last week of September 2021 increased 8.94% and 11.62% in case the government decided to enforce micro and macro quarantine. For the Omicron period, micro quarantine resulted 2.22% and 5.20% higher number of $D_{dm}$ and $C_{dm}$, respectively. When the macro quarantine option was selected, it was possible that the number of diabetic without complications increases 2.96% and the number of diabetic with complications increases 6.40% in the end of observation.

### 3.2. Effect of Tuberculosis and Diabetes Control Strategies during COVID-19 Quarantine

We proposed some control strategies in this work to reduce the risk of tuberculosis and diabetes during the implementation of quarantine that focus at mitigating the COVID-19 disease transmission. We added three control functions $\left(u_{1},u_{2},u_{3}\right)$ related to the reduction of infected tuberculosis, and two controls $\left(u_{4},u_{5}\right)$ intended to minimize the probability of developing diabetes. We assumed that the controls were continuous defined by a constant rate. Each control simulated both separately and combined. Here, the percentage of reduction in the total number of cases during observation time compared to no quarantine scenario was chosen to illustrate the efficacy of each control strategy.

**Figure 5:**
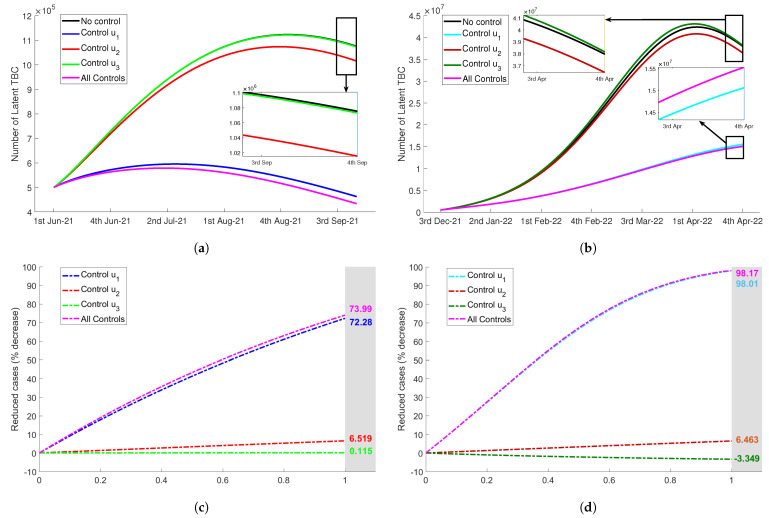
The impact of TB controls implementation $\left(u_{i}=0.5\right)$ to the number of latent people $L_{tb}$ during Delta period (**a**), and Omicron period (**b**). Next, the efficacy of tuberculosis controls with the variations of control value, $0\leq u_{1},u_{2},u_{3}\leq 1$ to reduce the number of latent $L_{tb}$ were shown in figure (**c**) for Delta and in figure (**d**) for Omicron. (**a**) The number of $L_{tb}$ during Delta period. (**b**) The number of $L_{tb}$ during Omicron period. (**c**) Controls efficacy reducing $L_{tb}$ during Delta period. (**d**) Controls efficacy reducing $L_{tb}$ during Omicron period.

**Figure 6:**
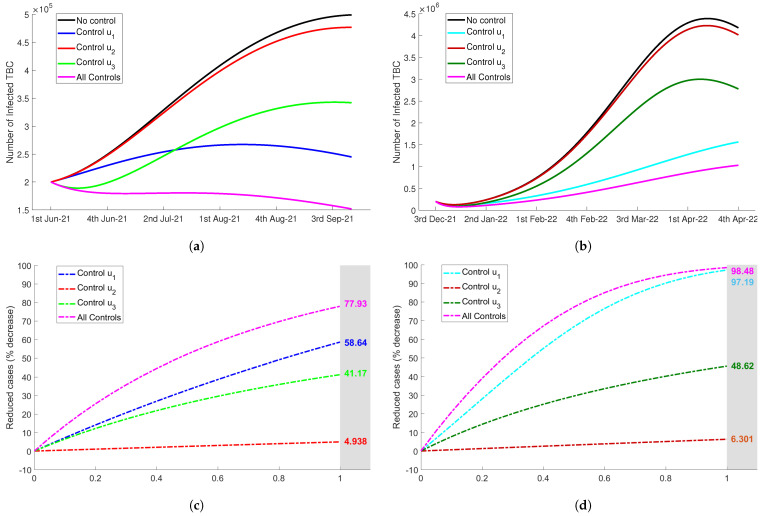
The impact of TB controls implementation $\left(u_{i}=0.5\right)$ to the number of infected people $I_{tb}$ during Delta period (**a**), and Omicron period (**b**). Next, the efficacy of tuberculosis controls with the variations of control value, $0\leq u_{1},u_{2},u_{3}\leq 1$ to reduce the number of infected $I_{tb}$ were shown in figure (**c**) for Delta and in figure (**d**) for Omicron. (**a**) The number of $I_{tb}$ during Delta period. (**b**) The number of $I_{tb}$ during Omicron period. (**c**) Controls efficacy reducing $I_{tb}$ during Delta period. (**d**) Controls efficacy reducing $I_{tb}$ during Omicron period.

For the first scenario, we used merely the control $u_{1}\left(t\right)$. This control intended to increase public awareness to protect them from tuberculosis risk during quarantine at home. The awareness program were carried out by direct campaigns or by using mass media and social media to inform the citizens about the dangers of TB transmission in the close environment when they stayed at home. Using constant rate $u_{1}=0.5$, Figure 5a,b display significant reduction of latent TB during Delta and Omicron period. The variations in the value of control $u_{1}$, ranging $0\leq u_{1}\leq 1$, showed the efficacy in reducing latent TB up to 72.28% for Delta variant and 98.01% for Omicron variant (see Figure 5c,d). Also, reduced cases can be seen in the number infected TB $\left(I_{tb}\right)$ during both Delta and Omicron variant outbreaks (see Figure 6a,b). In Figure 6c,d, we can see that the efficacy of control $u_{1}\left(t\right)$ to reduce the number of active infected TB during the pandemic of Delta and Omicron variant was up to 58.64% and 97.19%, respectively.

Next, we used only the control $u_{2}\left(t\right)$ for the second scenario. This control represented the proportion of latent individuals $L_{tb}$ that was identified and received medical treatment. The expansion of the screening test and diagnosis for latent TB or people at high infection risk could be adopted. In Figure 5a,b, using constant rate $u_{2}=0.5$, we observed that the decrease in the number of latent $L_{tb}$ in both periods were less significant than the first proposed TB control strategy. Now, by varying control rate value, $0\leq u_{2}\leq 1$, the percentage of reduced cases were only up to 6.519% for Delta and only up to 4.463% for Omicron, as can be seen in Figure 5b,d. In addition, control $u_{2}$ also was not more effective than $u_{1}$ in reducing the number of infected TB $\left(I_{tb}\right)$. In Figure 6c,d, the effectiveness measurement showed that the efficacy of this type of control was only up to 4.938% during Delta period and 6.301% during Omicron period.

The third control proposed to reduce tuberculosis risk was $u_{3}\left(t\right)$. In this strategy, the diagnosis program for the infected individuals was intensified. A specific team could be formed to continue TB diagnosis and treatment program even though COVID-19 was a priority during the pandemic. This control was focused on the diagnosis of infected, so the latent was not significantly reduced. Even, in the Omicron period there was no decrease in the number of latent TB.In Figure 5c, we observed that the number of latent decreased only up to 0.115%. Although control $u_{3}$ was not significantly affecting latent TB, this control has potential to reduce the number of infected TB quite notably. The efficacy of this control in reducing $I_{tb}$ was up to 41.17% for Delta period and 48.62% for Omicron period as shown in Figure 6, with the values of control $u_{3}$ ranging $\left[0,1\right]$.

For the last scenario of tuberculosis control strategy, we combined all proposed control, $u_{1}$, $u_{2}$, and $u_{3}$. We assumed that all controls were implemented with equal rate, that was, $u_{1}=u_{2}=u_{3}$. As expected, Figure 5 and Figure 6 show that this combination decreased the number of $L_{tb}$ and $I_{tb}$ more significant than three previous single controls. More precisely, the latent individuals decreased up to 73.99% and 98.17% during Delta and Omicron period. For the infected individuals, there were reductions by 77.93% and 98.48% during the observation time starting from June 2021 to September 2021 for Delta variant and from December 2021 to April 2022 for Omicron variant, respectively.

**Figure A1:**
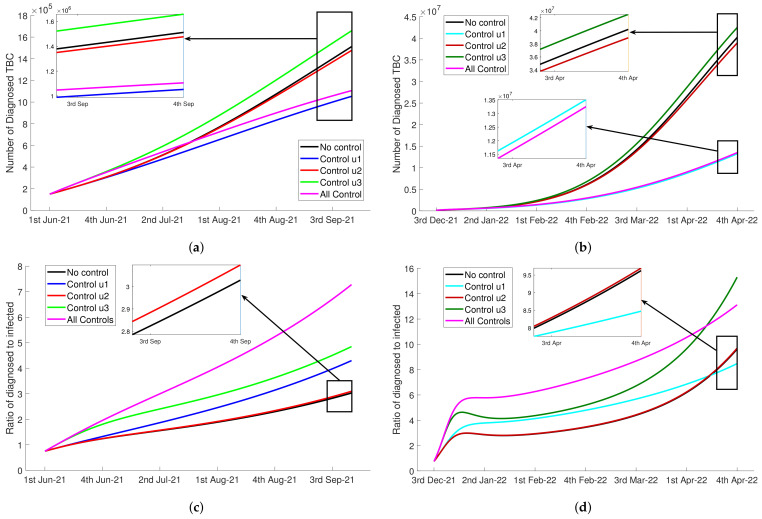
The impact of tuberculosis controls implementation to the number of diagnosed people $D_{tb}$ for Delta period (**a**), and Omicron period (**b**). The ratios of diagnosed people $D_{tb}$ over infected people $I_{tb}$ during quarantine during Delta and Omicron variant attacks were shown in figure (**c**) and (**d**), respectively. (**a**) The number of $D_{tb}$ during Delta period. (**b**) The number of $D_{tb}$ during Omicron period. (**c**) The ratio of $D_{tb}$ to $I_{t}b$ during Delta period. (**d**) The ratio of $D_{tb}$ to $I_{t}b$ during Omicron period.

One of the important indicators that need to be considered regarding the effectiveness of a TB control strategy was the ratio between $D_{tb}$ and $I_{tb}$. The high ratio of diagnosed who receive medical treatment from health services over the infected indicated that the strategy was more effective. In Appendix A, Figure A1 shows the number of $D_{tb}$ during the observation time, and we observed that the highest ratio of $D_{tb}$ over $I_{tb}$ was shown by the combination of all controls. The control $u_{3}$ significantly increased the ratio because it was focused on the increase of diagnosis rate. The control $u_{2}$, aimed to reduce the number of latent, showed low value of $D_{tb}/I_{tb}$ and did not significantly influence the diagnosis rate.

**Figure 7:**
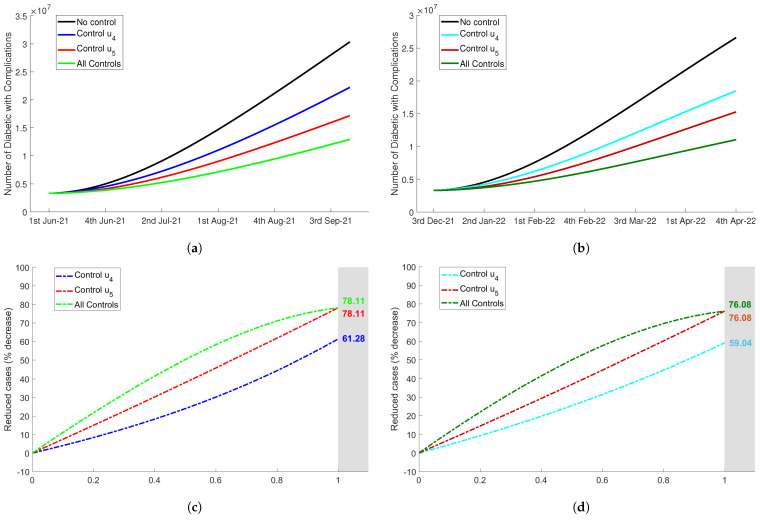
The impact of diabetes controls implementation to the number of diabetic people with complications $C_{dm}$, figure (**a**) for Delta period and figure (**b**) for Omicron period. The efficacy of diabetes controls to reduce the number of diabetics $C_{dm}$ with the variations of control value, $0\leq u_{4},u_{5}\leq 1$ were shown in figure (**c**) and (**d**) for Delta variant and Omicron variant, respectively. (**a**) The number of $C_{dm}$ during Delta period. (**b**) The number of $C_{dm}$ during Omicron period. (**c**) Controls efficacy reducing $C_{dm}$ during Delta period. (**d**) Controls efficacy reducing $C_{dm}$ during Omicron period.

For the first diabetes reduction scenario, we compared the number of diabetic with complications $\left(C_{dm}\right)$, with and without control $u_{4}\left(t\right)$. The goal of this strategy was to increase public awareness to implement healthy lifestyle, set a good diet, and exercise at home regularly in quarantine period. The implementation of constant control $u_{4}=0.5$ reduced the number of $C_{dm}$ as shown in Figure 7a,b. Figure 7c,d show that the upper bound of this control efficacy was equal to 61.28% and 59.04% for Delta and Omicron period, respectively. This implied a reduction in the number of people with diabetes of more than half of the total cases when control $u_{4}$ was not considered.

Next, we used only the control $u_{5}\left(t\right)$ to reduce the probability of diabetics developing complications during the implementation of quarantine. Diet tracking, regular diet, and healthy lifestyle in the quarantine period could be adopted to prevent the emergence of complications. In Figure 7a,b, we observed that the number of $C_{dm}$ reduced more significant than control $u_{4}$. By using constant rate $0\leq u_{5}\leq 1$ shown in Figure 7c,d, the percentage of reduced cases was up to 78.11% for Delta and 76.08% for Omicron.

In addition, we combined control $u_{4}$ and control $u_{5}$. In this strategy, the two controls were applied at the same time in order to obtain better numerical results. We assumed that the controls had equal rate, $u_{4}=u_{5}$. In Figure 7a,b, we used controls $u_{4}=u_{5}=0.5$ in the numerical simulation. The uppper bound of the efficacy of this control combination was equal to the upper bound of single control $u_{5}$, but lower values of this combination yielded higher efficacy than control $u_{5}$ as shown in Figure 7c,d.

**Figure A2:**
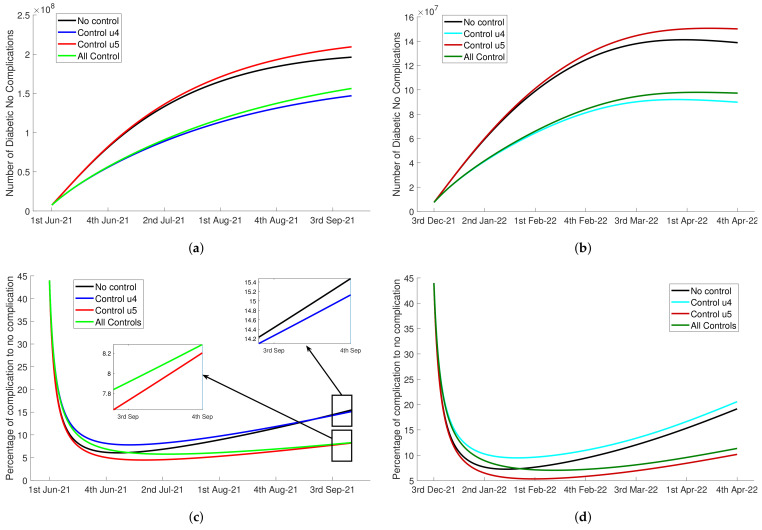
The impact of diabetes controls implementation to the number of diabetic people without complications $D_{dm}$ during Delta period shown in (**a**) and during Omicron period shown in (**b**). The percentage of diabetic people with complications $C_{dm}$ over the diabetic without complications $D_{dm}$ on Delta and Omicron quarantine were shown in figure (**c**) and (**d**), respectively. (**a**) The number of $D_{dm}$ during Delta period. (**b**) The number of $D_{dm}$ during Omicron period. (**c**) Percentage of $C_{dm}$ to $D_{dm}$ during Delta period. (**d**) Percentage of $C_{dm}$ to $D_{dm}$ during Omicron period.

In Appendix A, Figure A2 shows that control $u_{4}$ significantly reduced the number of diabetic without complications but this control did not detain the progression from $D_{dm}$ to diabetic with complications, $C_{dm}$. The implementation of control $u_{5}$, that was aimed to reduce the probability of developing complications, showed the low percentage of $D_{dm}$ becoming $C_{dm}$. The fatal impact of diabetes with complications could be minimized by implementing strategy $u_{5}$ during quarantine period. On the other hand, control $u_{5}$ was not effective to decline the number of diabetic without complications $D_{dm}$. The combination of control $u_{4}$ and $u_{5}$ could be considered as the best strategy to reduce the risk of diabetes development during quarantine implementation. These strategies decreased the number of $D_{dm}$ and $C_{dm}$, and reduced the possibility of $D_{dm}$ developing complications.

## 4. Discussion

In order to control the COVID-19 transmission during pandemic, numerous countries have decided to adopt lockdown and regional quarantine policies. It had been a considerable time since such strategies were last introduced, and currently they were implemented on global scale. Despite the fact that quarantines have been implemented relatively recently, some contributions have already appeared in the literature, aimed at evaluating how this policies work, along with its efficacy in terms of controlling virus infection. Lau et al. [16] have analysed the Wuhan case and have highlighted the significance of these measures to reduce the contagion probability by significantly decrease the growth rate and increase the doubling time of cases. Guzzetta et al. [17] have reported that national lockdown in Italy brought net reproduction ratio $\left(R_{t}\right)$ below 1 within 2 weeks and the epidemic was brought under control only after the implementation of lockdown. A similar finding was reported by Megarbane et al. [18] that lockdown and quarantine were considered as an effective intervention to halt coronavirus epidemic progression in nine different countries (UK, USA, Germany, Spain, New Zealand, Italy, France, Netherlands, and Sweden).

Delta (B.1.617.2) and Omicron (B.1.1.529) are two variants of SARS-CoV-2 that causes high infection during COVID-19 pandemic. The Delta variant was originally found in India in December 2020 and the Omicron variant was firstly observed in South Africa in the early days of November 2021 [19,20]. The Delta variant has spread over very fast because of its capability to invade the host’s immune system, but the Omicron variant has been reported to be more infectious than previous variants with a short doubling time. In this work, we found that the infection rates of Omicron variant, $\left(\beta_{1},\beta_{2},\epsilon_{1},\epsilon_{2}\right)$, were approximately 1.04–1.26 times higher than the infection rates of Delta variant. This finding is in accordance with the results of research conducted by Lyngse et al. [21] in Denmark that the rate of Omicron virus infection was 1.17 times higher than Delta variant, especially for unvaccinated people. Chaguza et al. [22] also reported that Omicron variant is 1.3 times more infectious than Delta due to the increased transmission acquired from the mutation.

The implementation of lockdown and regional quarantine to control COVID-19 pandemic have influenced tuberculosis clinical management and health services related to TB. In the countries and territories where healthcare personnel assigned in TB programs have been diverted to handle COVID-19 patients, the impact of pandemic on tuberculosis treatments is estimated to be severe. Numerous countries have ruled out TB programs because COVID-19 control programs are priority and urgently needed. Health facilities throughout the country become the battleground for COVID-19. To decrease the potential risk of viral transmission to either health care workers or patients during their visits, hospitals are minimizing the number of daily outpatient visits. Several health services are prepared to meet the demands of the overwhelming number of COVID-19 patients.

Migliori et al. [23] have reported that COVID-19 pandemic has interfered TB-related services globally. Data from 33 TB centers in 16 countries indicated the reductions in the diagnosis of newly active TB and total outpatient visits of active or latent TB during lockdown and regional quarantine in the first 4 months of 2020. The study by Lange et al. [24] showed a significant decline in emergency department visits such as TB centers, suggesting that patients may be avoiding care or unable to access care during the pandemic. The reduction in the number of outpatient visits may be due to the patient’s fear of exposure severe acute respiratory syndrome COVID-19 [25]. Because of lockdown and quarantine, progression to active TB from latent TB who did not obtain preventive measure and medical treatment was possibly occurred [26]. Despite the number of outpatient TB visits decreased, the implementation of lockdown and regional quarantine have increased the interest to use telemedicine. TB programs offered new service called telehealth. The use of telehealth services in the United Kingdom, India, Russia, and Australia was considerably higher in 2020, driven by social distancing policies and in accordance with the innovation program to answer challenges during the pandemic [27,28]. In Indonesia, some examples of telehealth services are https://www.temenin.kemkes.go.id (accessed on 14 November 2022) provided by Indonesian Health Ministry and Halodoc, an application developed by PT Media Dokter Investama. These services bring together patients with expert doctors for online consultations, diagnosing the patient’s condition, and providing medicine recommendations or other medical treatments.

In tuberculosis epidemiology, the duration and proximity of exposure to an active TB as the source of infection led to an increased risk of TB infection. It should be noticed that the implementation of lockdown and regional quarantine may possibly increase the risk of TB infection during COVID-19 pandemic. The government enforced stay-at-home policies and confined the citizens in their family environment. In the family-household setting, a persistent close contact with family members suffering from active TB and still undiagnosed increased the transmission risk to susceptible persons [29]. The prevention method and awareness program should be adopted, through the campaign of awareness directly or indirectly using mass media or social media to inform the citizens about the dangers of TB transmission in the close environment when they stay at home. By taking preventive measures such as applying health lifestyle, covering coughs and sneezes, and keeping hands clean, the risk of TB infection could be reduced. Family members who show TB symptoms should immediately visit the health service or contact the health care by using telehealth facility to avoid wider transmission caused by undetected case.

The change in nutritional habits and lifestyle are unavoidable negative consequences of lockdown and quarantine. Limited access to food and reduced availability of goods caused by restricted opening hours of store lead to the changing in nutritional habits and the switching to unhealthy food. A recent review by Brooks et al. [30] reported negative psychological effects of quarantine including stress and anxiety. Mental health issues such as stress and anxiety are considered to be associated with unhealthy lifestyle that drive people to eat and drink in an attempt to get better feeling. Eating unhealthy foods regularly such as snacks, chocolates, junk foods, fast foods, and soda cola, and drinking spirits and wine more frequently are more likely to be new habits of these stress-driven eaters and drinkers. This leads to weight gain that may possibly contributes to development of diabetes. In order to reduce diabetes risk as the side effect of the implementation of lockdown and quarantine, a healthy diet should be applied. Vegetarian diet and Mediteranian diet are the examples of healthy diet that give important metabolic advantages for preventing and treating diabetes and its complications [31,32].

Other than the unhealthy eating habits, the reduction of physical activity also contributes the weight gain in quarantine period. Pandemic-related closure of public exercise facilities such as sports centers, gymnasiums, and swimming pools may disproportionately influence active individuals. Notwithstanding the guidances to exercise at home, only few citizens comply. Regular physical activity is mandatory to maintain health status. The human body’s metabolism is strongly influenced by physical activity. Doing more physical activity is one important factor to lowering the risk of diabetes because it decreases the glucose level in blood circulation by increasing glucose uptake [33,34]. Promotion of physical activity in home needs to be intensified during the implementation of lockdown and regional quarantine. In addition, wherever and whenever possible and allowed, while following the government rules, people should be suggested to be more active outdoors, preferably in green open space. As in all other situations, regulations of wearing mask and social distancing are also essential in outdoors to reduce the COVID-19 infection risk.

## 5. Conclusions

In this paper, we proposed a mathematical model and used numerical simulation to describe the impact of quarantine on tuberculosis and diabetic people during COVID-19 pandemic. A compartmental nonlinear deterministic epidemic model, including three diseases: COVID-19, tuberculosis, and diabetes, was formulated. We aimed to point out the potential negative effects, particularly on tuberculosis and diabetic people, when the government implemented isolation measures such as lockdown and regional quarantine. The mathematical model fitted with the actual data of COVID-19 cases in Indonesia when the Delta and Omicron variants were identified. We also suggested some control strategies to reduce the negative impact in both tuberculosis and diabetic people during quarantine. The results of numerical simulation indicate different effectiveness and efficacy of each control strategy. By increasing public awareness about the dangers of TB transmission in environment when they stayed at home, the number of newly infected TB during quarantine can be reduced significantly. In addition, in order to minimize the risk of diabetes progression with or without complications, the implementation of healthy lifestyle and exercise inside the house are considered as the most effective strategy.

This study has several limitations that can be developed for future research. The actual problem is complex and this study aims to simplify it. Therefore, the mathematical model that we developed only consists of the basic compartments and variables of the COVID-19 disease that we consider essential. Immunity level due to vaccination, time between infection and the acute phase of disease, symptomatic or asymptomatic infected individuals, and other important variables can be taken into account in further studies. The second limitation is the realtionship between TB and diabetes mellitus is not accommodated in the mathematical model, even though diabetes is an important factor and a comorbid of TB. Thirdly, the availability of TB and diabetes data during the COVID-19 pandemic is potentially improve the output of model, where the results of the data fitting are not only in accordance with the COVID-19 data but also the TB and diabetes data. In our study, we were unable to obtain the proper TB and diabetes due to the lack of data recording.

## Figures and Tables

**Table 1:** The variations of quarantine rate value based on the level of quarantine.

Parameter	Description	Level of Quarantine		
		No	Micro	Macro
$\theta_{1}$	Quarantine rate from susceptible to quarantined susceptible	0.00	0.30	0.75
$\theta_{2}$	Quarantine rate from infected coronavirus to quarantined infected	0.95	0.85	0.75

**Table 2:** Description of parameters used in mathematical model with estimated value.

Parameter	Description	Delta (95% CI)	Omicron (95% CI)
$\beta_{1}$	Infection rate of susceptible by contact with infected	4.411(4.077,4.745)	5.569(5.313,5.825)
$\beta_{2}$	Infection rate of susceptible by contact with quarantined infected	6.125(5.671,6.580)	6.371(6.001,6.742)
$\epsilon_{1}$	Infection rate of quarantined susceptible by contact with infected	3.595(3.414,3.776)	4.429(4.372,4.486)
$\epsilon_{2}$	Infection rate of quarantined susceptible by contact with quarantined infected	5.985(5.528,6.442)	5.129(4.763,5.494)
$\rho_{1}$	Infection rate of susceptible by contact with infected TB	0.335(0.291,0.378)	1.884(1.624,2.144)
$\rho_{2}$	Infection rate of susceptible by contact with diagnosed TB	0.349(0.307,0.391)	1.628(1.302,1.954)
$\tau_{1}$	Infection rate of quarantined susceptible by contact with infected TB	0.486(0.426,0.545)	2.101(1.869,2.333)
$\tau_{2}$	Infection rate of quarantined susceptible by contact with diagnosed TB	0.492(0.436,0.548)	2.839(2.672,3.007)
$\phi_{2}$	Diagnosis rate of infected TB	0.497(0.438,0.555)	2.237(1.905,2.569)
$\delta_{1}$	Probability of susceptible developing diabetes	0.091(0.084,0.098)	0.067(0.062,0.072)
$\delta_{2}$	Probability of quarantined susceptible developing diabetes	0.129(0.121,0.136)	0.085(0.076,0.093)

**Table 3:** The summary of COVID-19 infected people, $I_{co}+Q_{2}$, in three scenarios of quarantine.

Variant	Indicator	No Quarantine	Micro Quarantine	Macro Quarantine
Delta (B.1.617.2)	Total infected individuals in 17 weeks	6,691,270	3,246,557	2,397,179
	Peak of infection	4th week of July 2021	3rd week of July 2021	2nd week of July 2021
	Highest potential number of cases	734,323	386,249	288,169
	Number of cases at the end of observation	117,673	16,968	9201
	Percentage of reduced cases	-	51.48%	64.17%
Omicron (B.1.1.529)	Total infected individuals in 19 weeks	11,188,961	3,383,183	2,282,320
	Peak of infection	1st week of March 2022	4th week of February 2022	3th week of February 2022
	Highest potential number of cases	1,382,465	426,239	286,566
	Number of cases at the end of observation	88,804	4155	2021
	Percentage of reduced cases	-	69.76%	79.60%

**Table 4:** The number of infected tuberculosis and diabetic people in the end of observation time.

Variant	Compartment	No Quarantine	Micro Quarantine	Macro Quarantine
Delta (B.1.617.2)	Infected tuberculosis $\left(I_{tb}\right)$	$4.39\times 10^{5}$	$4.97\times 10^{5}$	$5.01\times 10^{5}$
	Diagnosed tuberculosis $\left(D_{tb}\right)$	$1.38\times 10^{6}$	$1.49\times 10^{6}$	$1.51\times 10^{6}$
	Diabetes without complications $\left(D_{dm}\right)$	$1.77\times 10^{8}$	$1.93\times 10^{8}$	$1.96\times 10^{8}$
	Diabetes with complications $\left(C_{dm}\right)$	$2.72\times 10^{7}$	$2.96\times 10^{7}$	$3.03\times 10^{7}$
Omicron (B.1.1.529)	Infected tuberculosis $\left(I_{tb}\right)$	$3.04\times 10^{6}$	$4.14\times 10^{6}$	$4.18\times 10^{6}$
	Diagnosed tuberculosis $\left(D_{tb}\right)$	$2.59\times 10^{7}$	$3.81\times 10^{7}$	$4.02\times 10^{7}$
	Diabetes without complications $\left(D_{dm}\right)$	$1.35\times 10^{8}$	$1.38\times 10^{8}$	$1.39\times 10^{8}$
	Diabetes with complications $\left(C_{dm}\right)$	$2.50\times 10^{7}$	$2.63\times 10^{7}$	$2.66\times 10^{7}$

## Data Availability

The data presented in this study are available on request from the corresponding author.

## Appendix A

### Appendix A.1

**Table A1:** Description of parameters used in mathematical model with fixed value.

Parameter	Description	Value	Unit	References
$\mu_{h}$	Human natural birth or mortality rate	1/(65 × 52)	week${}^{-1}$	[35,36,37]
α	Rate of progression to infected from exposed	7/6.5	week${}^{-1}$	[38,39,40]
$d_{1}$	Mortality rate caused by COVID-19 infection	0.00039	week${}^{-1}$	data
γ	Recovery rate of infected and quarantined of COVID-19	7/3.6	week${}^{-1}$	[40,41,42]
$\phi_{1}$	Rate of progression to infected from latent	7/60	week${}^{-1}$	[43,44]
$\gamma_{1}$	Recovery rate of latent tuberculosis	0.01285	week${}^{-1}$	[43,44]
$\gamma_{2}$	Recovery rate of infected tuberculosis	0.00122	week${}^{-1}$	[43]
$\gamma_{3}$	Recovery rate of diagnosed tuberculosis	0.00764	week${}^{-1}$	[43]
$d_{2}$	Mortality rate caused by tuberculosis infection	0.00111	week${}^{-1}$	[45]
ν	Probability of diabetic people developing complications	0.01303	week${}^{-1}$	[46]
ω	Probability of diabetic people recovered from complications	0.00714	week${}^{-1}$	[46]
$d_{3}$	Mortality rate caused by diabetes mellitus	0.00013	week${}^{-1}$	[46]

### Appendix A.2

The resulting states completed by the controls for each compartment are given by the following equations:

$$\begin{array}{cc} \; & \frac{dQ_{1}}{dt}=\theta_{1}S-\left(\frac{\epsilon_{1}I_{co}+\epsilon_{2}Q_{2}}{N}\right)Q_{1}-\left(1-u_{1}\left(t\right)\right)\left(\frac{\tau_{1}I_{tb}+\tau_{2}D_{tb}}{N}\right)Q_{1}-\left(\left(1-u_{4}\left(t\right)\right)\delta_{2}+\mu \right)Q_{1}\\ \; & \frac{dL_{tb}}{dt}=\left(\frac{\rho_{1}I_{tb}+\rho_{2}D_{tb}}{N}\right)S+\left(1-u_{1}\left(t\right)\right)\left(\frac{\tau_{1}I_{tb}+\tau_{2}D_{tb}}{N}\right)Q_{1}-\left(\phi_{1}+\left(1+u_{2}\left(t\right)\right)\gamma_{1}+\mu \right)L_{tb} \end{array}$$

$$\begin{array}{cc} \; & \frac{dI_{tb}}{dt}=\phi_{1}L_{tb}-\left(\left(1+u_{3}\left(t\right)\right)K\phi_{2}+\gamma_{2}+d_{2}+\mu \right)I_{tb}\\ \; & \frac{dD_{tb}}{dt}=\left(1+u_{3}\left(t\right)\right)K\phi_{2}I_{tb}-\left(\gamma_{3}+d_{2}+\mu \right)D_{tb}\\ \; & \frac{dR_{tb}}{dt}=\left(1+u_{2}\left(t\right)\right)\gamma_{1}L_{tb}+\gamma_{2}I_{tb}+\gamma_{3}D_{tb}-\mu R_{tb}\\ \; & \frac{dD_{dm}}{dt}=\delta_{1}S+\left(1-u_{4}\left(t\right)\right)\delta_{2}Q_{1}+\omega C_{dm}-\left(\left(1-u_{5}\left(t\right)\right)\nu +\mu \right)D_{dm}\\ \; & \frac{dC_{dm}}{dt}=\left(1-u_{5}\left(t\right)\right)\nu D_{dm}-\left(\omega +d_{3}+\mu \right)C_{dm} \end{array}$$

where the remaining states did not change.
